# Complete Nucleotide Sequence of an Australian Isolate of *Turnip mosaic virus* before and after Seven Years of Serial Passaging

**DOI:** 10.1128/genomeA.01269-16

**Published:** 2016-11-17

**Authors:** Lara Pretorius, Richard L. Moyle, Jessica Dalton-Morgan, Nasser Hussein, Peer M. Schenk

**Affiliations:** Nexgen Plants Pty. Ltd. and School of Agriculture and Food Sciences, The University of Queensland, Brisbane, Queensland, Australia

## Abstract

The complete genome sequence of an Australian isolate of *Turnip mosaic virus* was determined by Sanger sequencing. After seven years of serial passaging by mechanical inoculation, the isolate was resequenced by RNA sequencing (RNA-Seq). Eighteen single nucleotide polymorphisms were identified between the isolates. Both isolates had 96% identity to isolate AUST10.

## GENOME ANNOUNCEMENT

*Turnip mosaic virus* is a *Potyvirus* member within the *Potyviridae* family. Due to the wide distribution and extensive host range, *Turnip mosaic virus* (TuMV) is considered to be the second most damaging crop virus across 28 different countries and regions (1). TuMV virions are flexuous filaments of approximately 700 to 750 nm in length. TuMV has a single-strand positive-sense RNA genome, approximately 10 kb in length, encoding a single open reading frame flanked by two untranslated regions. The translated polypeptide is cleaved and processed into 10 viral proteins.

Here, we report the complete sequence of an Australian isolate of TuMV, designated TuMV-QLD1a, from a field-grown *Brassica pekinensis* plant from Toowoomba, Queensland, in 1997 (Department of Agriculture and Fisheries plant virus collection accession no. VIR0745). RNA was isolated from mechanically inoculated *Nicotiana benthamiana* and cDNA synthesized as previously described (2). Ten overlapping fragments were PCR amplified and Sanger sequenced with at least two-fold coverage in both the forward and reverse orientation. The TuMV isolate was subsequently resequenced after being passaged over a seven-year period by serial mechanical inoculation of *N. benthamiana* seedlings every two to three months. The resequenced isolate, designated TuMV-QLD1b, was resolved by RNA sequencing (RNA-Seq) analysis using the TruSeq RNA library synthesis kit (Illumina) and the MiSeq platform (Illumina). After quality trimming, 33,752,578 RNA-Seq reads were obtained, and the Geneious version 8.1.7 software was used to map 1,482,837 reads to the TuMV-QLD1a reference genome. The average depth of coverage was 15,905×, with maximum coverage of 26,792×, and minimum coverage of 578×.

Both TuMV-QLD1a and TuMV-QLD1b are 9,796 nucleotides (nt) in length. However, 18 single nucleotide polymorphisms (SNPs) were identified between the two isolates. Of these, two were heterogeneous SNPs in TuMV-QLD1a that were found to be uniform in TuMV-QLD1b (A/G→A and A/G→G). Conversely, five different heterogeneous SNPs emerged in TuMV-QLD1b. An amino acid alignment revealed 10 amino acid changes from TuMV-QLD1a to TuMV-QLD1b. Both isolates share 96% identity with the AUST10 isolate (accession number AB989634), which was also collected in Queensland, Australia, in 1996 (3). Phylogenetic analysis places TuMV-QLD1a and TuMV-QLD1b in the basal-B group and subgroup basal-B2. This group is unable to infect *Raphanus* plants but does infect *Brassica* plants systemically causing phenotypic symptoms (4). This is considered to be the most variable group, as it emerged paraphyletic to the other lineages (5). This subgroup is most closely related to four German isolates, DEU7, AIIA, TIGD, and TIGA (accession numbers AB701695.1, AB701694.1, AB701735.1, and AB701734.1, respectively) and is thought to have been introduced into Australia and New Zealand via horticultural material (3). DEU7 and AIIA have 87% identity to both versions of TuMV-QLD1, while TIGD and TIGA have 86% identity.

### Accession number(s).

The GenBank accession numbers for TuMV-QLD1a and TuMV1b are KX641465 and KX641466, respectively.

## References

[1] Walsh JA, Jenner CE 2002 Turnip mosaic virus and the quest for durable resistance. Mol Plant Pathol 3:289–300. doi:10.1046/j.1364-3703.2002.00132.x. 20569337

[2] Moyle R, Pretorius L, Dalton-Morgan J, Persley D, Schenk P 2016 Analysis of the first complete genome sequence of an Australian tomato spotted wilt virus isolate. Australasian Plant Pathol 45:509–512. doi:10.1007/s13313-016-0435-2.

[3] Yasaka R, Ohba K, Schwinghamer MW, Fletcher J, Ochoa-Corona FM, Thomas JE, Ho SY, Gibbs AJ, Ohshima K 2015 Phylodynamic evidence of the migration of turnip mosaic potyvirus from Europe to Australia and New Zealand. J Gen Virol 96:701–713. doi:10.1099/jgv.0.000007. 25481753

[4] Tomimura K, Gibbs AJ, Jenner CE, Walsh JA, Ohshima K 2003 The phylogeny of turnip mosaic virus; comparisons of 38 genomic sequences reveal a Eurasian origin and a recent “emergence” in East Asia. Mol Ecol 12:2099–2111. doi:10.1046/j.1365-294X.2003.01881.x. 12859632

[5] Nguyen HD, Tran HT, Ohshima K 2013 Genetic variation of the turnip mosaic virus population of Vietnam: a case study of founder, regional and local influences. Virus Res 171:138–149. doi:10.1016/j.virusres.2012.11.008. 23201192

